# Students’ Perceived Well-Being and Online Preference: Evidence from Two Universities in Vietnam during COVID-19

**DOI:** 10.3390/ijerph191912129

**Published:** 2022-09-25

**Authors:** Nam Hoang Tran, Nhien Thi Nguyen, Binh Thanh Nguyen, Quang Ngoc Phan

**Affiliations:** 1 Research Center for Higher Education, Tokushima University, Tokushima 770-8502, Japan; 2 Faculty of Biotechnology, Vietnam National University of Agriculture, Hanoi 131000, Vietnam; 3 Department of Scientific Management, Thai Binh University of Medicine and Pharmacy, Thai Binh 410000, Vietnam; 4 The Center Service for Technology Science of Medi-Phar., Thai Binh University of Medicine and Pharmacy, Thai Binh 410000, Vietnam

**Keywords:** COVID-19, impact, online education, satisfaction, support, Vietnam, well-being

## Abstract

University education is still being impacted two years after the COVID-19 outbreak. We performed a rapid survey in February 2022 at two public universities in Vietnam to examine the effects of the pandemic on well-being and the factors that may associate with online class preference among university students as well as to investigate the need for support to improve resilience. A web-based survey included 1589 undergraduate students in total. Both quantitative and qualitative data analysis was carried out. Overall, approximately a quarter of respondents said that they perceived an influence on their health, 42.9% expressed stress, and more than 70% reported worrying about the future. In total, 61.9% of the respondents reported having satisfaction with online classes, while over half of them preferred a program of 50% online classes. Students who live in an urban area, are female, have had pre-COVID-19 campus life experience, have decreased income, and/or experience low online satisfaction and over-information may be in need of more support. The results show implications for universities to consider policies addressing well-being and post-pandemic online education. Providing support to university students to improve their resilience against the impact on their studying, campus life, health, and well-being should be prioritized during and post-pandemic.

## 1. Introduction

Higher education provides the environment for students to achieve their academic performance and experience new life events. Campus life is also a place that may inflict impacts on students’ health and well-being, which has been becoming a subject of extensive studies [1,2,3,4,5]. Among students in various undergraduate courses, students in medicine, veterinary, and some similar disciplines are required to study for longer years and with more practical training. Studies have demonstrated that medical students are more prone to health and well-being problems than other undergraduate student groups [6,7].

The emergence of the COVID-19 pandemic and subsequent disruptions in higher education brought additional factors and impacts to students’ well-being as well as brought a new perspective about online education as an alternative to the traditional face-to-face classroom. In the post-pandemic era, the higher education sector is expected to face challenges, transformation, and reforms [8,9,10]. E-learning and digital transformation in higher education were introduced a long time ago, but they did not achieve widespread use and popularity until during or after the outbreak of the COVID-19 pandemic. Although it has pros and cons, online education has become a learning environment preferred by university students, witnessing a shift toward informal and flexible learning environments [11]. The rise of online education and distance learning seems to be inevitable in the future of global higher education [12].

Early effects of the COVID-19 pandemic were being felt in Asian nations. Studies in China, Korea, India, Indonesia, and the Philippines have shown that university students in Asian countries have been impacted by measures such as digital transformation, social distancing, and lockdowns [13,14,15]. Studies have also shown how exposure to COVID-19 affects students’ health and well-being, as well as their academic performance and day-to-day activities, and what needs to be addressed. For instance, a study conducted in the Asia Pacific region revealed that university students who struggle with online learning and job search are among the population groups most susceptible to psychological problems [16]. Moreover, excessive exposure to information on COVID-19 is linked to the development of psychiatric issues [17].

One of the first and worst-hit COVID-19 countries in Asia is Vietnam, a developing nation in Southeast Asia. Despite effective efforts to suppress the pandemic for the majority of 2020, it has since April 2021 been undergoing an uncontrolled resurgence epidemic [18]. The early episodes of national lockdown and social distancing have led to an impact on higher education. Although Vietnam has long been influenced by COVID-19, little evidence has been found about its impact on higher education. After the epidemic began, a survey carried out in 12 universities revealed varied difficulties and support for online education in Vietnam [19,20]. Another survey conducted on 1875 college students in Vietnam reported that negative moods were perceived in a quarter of respondents, while over 60% did not think online education would become the upcoming trend [21]. Another survey conducted in October 2021 at Vietnam National University Ho Chi Minh found that students have had common mental health problems during the COVID-19 period, such as sleep disorders, mood changes, sadness, and anxiety [22].

All the studies above were conducted at the beginning or after a year since the outbreak. We made the decision to conduct a quick web survey at two national universities in Vietnam in order to examine the effects of the COVID-19 pandemic two years after the breakout on students’ perceptions of their health and the quality of campus life. We choose two major public universities in Northern Vietnam. The first site—Thai Binh University of Medicine and Pharmacy (hereafter, TBUMP), established in 1968, is a public university located in Northern Vietnam, hosting over 7000 students majoring in medicine, pharmacy, and other health-care-related specialties [23]. The second survey site is the National University of Agriculture (hereafter, VNUA), established in 1956, and is a large-scale public university with over 20,000 students majoring in agriculture-related fields [24]. Comparatively, VNUA is larger and located in a more urban area than the TBUMP.

Previously, we have explored the interrelation between the effect of the pandemic on students’ well-being, online class preference, and the potentially associating factors. In a small-scale study conducted on international students in Japan in early 2021 [25], we have found an association between the perceived impact on taking the class and online class satisfaction and that acceptance of online learning could be associated with factors such as enrollment status, living status, language proficiency, and access to information. In later reports, we also reported the association between online class satisfaction and perceived impact on health, stress, and worry, which may vary by country, university and individuals, year of enrollment, dormitory status, income change, and life plan change [26,27].

Two years after the COVID-19 pandemic’s onset, this article seeks to examine the effects of the pandemic on health and well-being as well as the variables that might be related to a preference for online classes. As a way to lessen any effects, it also looks into the necessity of providing support for their students. Our research questions are:

- Has the COVID-19 epidemic had a negative influence on students’ health, notably in terms of reported stress, perceived health impact, and perceived future worries?
- How would students prefer online classes in the future, and what factors are associated with this preference?
- What kind of support is needed for students to reduce the impact?

This study will bring updated data about the longer impacts of COVID-19 on university students at the time of two years after the outbreak and bring up implications for university managers and educators to consider strategies addressing well-being and post-pandemic online education, particularly for settings in developing countries.

## 2. Methods

### 2.1. Participants

The survey was conducted on a group of 1589 undergraduate students who were majoring in agriculture at VNUA and medicine at TBUMP (Table 1). The age of the respondents was assumed to be from 18 for first-year students to 23 for sixth-year students. Of all respondents, 1120 were female (70.1%), and 478 were male (29.9%).

### 2.2. Questionnaire

This study applied a mixed research method, as the quantitative data were intended to address the research questions about the influence of the COVID-19 pandemic on students’ well-being and online classes preference, and the qualitative data were intended to address the research question about the need for support, as well as to supplement the quantitative data. Google Forms was used to create a cross-sectional online survey questionnaire. For quantitative data, the questionnaire had four-level Likert-style questions, and for qualitative data, it had open-ended inquiries. The survey was created based on the data collection forms that were utilized in several small-scale earlier studies [25,26,27] with some adaptation to the current study’s context. The question consisted of four parts: (1) Personal Information; (2) Preference for online class; (3) Perceived impacts on academic life, including perceived impact on taking class, perceived impact on doing research, perceived impact on daily life, income change, life plan change, access to information on COVID-19; and (4) Perceived impacts on health and well-being, which consisted of perceived impact on health, perceived stress, and perceived worry for future. It took about 6 min to complete the questionnaire.

### 2.3. Data Collection

We conducted a cross-sectional study from 1 to 15 February 2022, right at the time of the fifth wave of COVID-19 in Vietnam. The weblink to the online survey questionnaire was created by using Google Forms. Participants were recruited by snowball sampling technique. Initially, a research invitation was delivered to a core group of students in several classes. They were encouraged to access the survey link and share it with the other students from the same university. Before respondents choose whether or not to participate, the survey’s goal was laid out on the questionnaire’s first page. Participation in this study was entirely voluntary. This study was conducted according to the guidelines of the Helsinki Declaration. Prior to taking part in the study, all participants were fully informed of the confidentiality policy. Since there was no personal information recorded, all participation information was completely anonymous. No incentives were provided to participants, and they could withdraw from the survey at any time.

### 2.4. Data Analysis

The data was exported to Excel format and was analyzed by using SPSS Statistics version 27.0 for Windows (IBM Corp., Armonk, NY, USA). Qualitative data were extracted from the open-ended responses, interpreted, and summarized using thematic analysis [28]. The word cloud was created using a free online tool [29]. Based on the associations among variables found within this data set, we proposed a conceptual framework for the relationship between individual factors such as university, gender, living status, and factors related to impacts on well-being, factors related to impacts on campus life, and factors related to online class preference. After grouping the variables that seemed closely associated, we performed Exploratory Factor Analysis (EFA) to check the validity of the relationships among factors in the framework.

## 3. Results

### 3.1. Respondents’ Characteristics

According to gender, enrollment status, year of enrollment, foreign student status, living status, and living place status, Table 1 displays the characteristics of the respondents from each university.

When looking at the respondents’ enrollment years, the fourth and fifth years have the most respondents. In this study, we pay attention to the differences in the impact of the COVID-19 pandemic and taking an online class by the status of pre-COVID-19 campus experience of the students. The first- and second-year students (20%) were the group of students who had not experienced pre-pandemic academic life because this survey was conducted roughly two years after the pandemic was announced. The third-year students were enrolled since September 2019 and had been experiencing a few months of pre-COVID-19 campus life, so we put the third-year students and older in the group having pre-COVID-19 campus experience. Only 3.8% of the respondents, mostly from Laos and Cambodia, were international students. In terms of their living arrangements, more than a third of respondents lived alone, and more than 40% shared a room. Only a fifth of the respondents lived with their families. The majority of respondents—about two-thirds—lived in rented rooms, while the remaining lived in university dormitories or their own homes.

### 3.2. Students’ Perceived Impacts on Academic Life and Well-Being

In Table 2, we show the percentage of students who perceived impact on health, stress, and worry for the future, comparing the younger group who has no pre-COVID-19 campus experience with the rest using the Pearson Chi-Square test. In total, about a quarter of students reported perceiving impact on health, including 22.3% of the students who perceived some impact on health, while 2.5% perceived a lot of impacts. Similarly, 42.9% of students reported perceiving stress, including 37.4% of the students who perceived some stress, while 6.5% perceived a lot of stress. Regarding perceived worry for the future, over 70% of students reported having worries about the future, including over a fifth with a lot of worries. The results revealed that, when compared to students who had no pre-COVID-19 campus experience, students with pre-COVID-19 campus experience tended to report a higher impact on their health and more stress. There was no significant difference in the perceived worries between the two groups.

In Table 3, we show the percentage of students who reported satisfaction with online classes, comparing the group who has no pre-COVID-19 campus experience with the rest using the Pearson Chi-Square test. In total, 61.9% of the students reported having some satisfaction with online classes, including 7.8% have shown high satisfaction. Regarding their opinion about the optimal proportion between online and face-to-face classes, over half of the respondents expressed a preference for 50% for online classes. In relation to their pre-COVID-19 campus experience, the results showed that students with pre-COVID-19 campus experience tended to show higher satisfaction with online classes than the students who had no such an experience. This group also shows a higher preference for 50% online classes, 80% online classes, and 100% online classes.

To investigate factors that may associate with the perceived impact on the well-being of the respondents, we performed the Pearson Chi-Squared test for the independent nominal variables, including university, foreign student, female, year of enrollment, pre-COVID-19 campus experience, living status, living place; and the Spearman correlation test for the ordinal variables with or without linearity (Table 4). We found a significant correlation between several factors and the well-being of respondents. In comparison to students from TBUMP, students from VNUA tended to experience stress and worry about the future more. International students tended to perceive less impact on health and worry for the future than local students. Being female was a factor associated with higher perceived stress and worry for the future. Year of enrollment was associated with perceived health impact and perceived stress. Students who had pre-COVID-19 campus experience (third year and above) tended to perceive less impact on health and less stress. The living alone status was found to be associated with higher perceived health impact and higher stress. The perception of a greater impact on well-being was generally higher when taking classes and conducting research were considered. High satisfaction with online classes and a higher preference for online classes might be associated with a lower impact on well-being. Students saw a greater impact on their well-being when they reported a stronger impact on eating, shopping, daily life, decreased income and increased income, and changing their life plans. Accessing more information on COVID-19 was associated with higher perceived stress and worry for the future. No significant correlation was found between the living in a dormitory status concerning the perceived impact on well-being.

### 3.3. Results of Qualitative Data on COVID-19 Impacts on Students’ Campus Life

The responders were questioned about how the pandemic had impacted their health. The responses may generally be divided into two categories: effects on physical and mental health. In terms of physical well-being, responses were usually: “I gained weight because of immobility”, “I feel bad because I can’t eat as I want, I lost appetite”, “I feel physically weak because of lacking exercise”, “I was frequently becoming sick”, “No outdoor activities makes me tired”, “I had to stay in hospital for quarantine because I was in close contact with an infected person, I was worried about being late to submit my assignment”, “I suffered from vaccine side effects”, “I had hair loss due to vaccination”, “My eyes got bad”, “I got back pain”, “Laying down too much makes my neck stiff“, “I had symptoms such as fever, cough, sore throat”, “I got acne from wearing a mask”, and “Wearing mask makes me feel difficult to breath”. Respondents spoke about mood swings, depression, tension, and worry in relation to mental health issues, which led to a protracted period of social withdrawal: “I feel depressed”, “I always worry about being infected”, “I can’t go to clinics for checking up other diseases”, “I become lazy and lack of motivation”, “It changed my biorhythm”, “My mental health is strongly affected because of lacking communication with people”, and “I feel sad because I cannot meet my friends”. Some responses, though, did not appear to be affected: “I am not affected much”.

Responses to questions regarding life changes and concerns about the future can be divided into three categories: employment, academics, and living in the future. Concerning academic issues, some respondents said: “By studying online, I feel bored and tired, I can’t concentrate”, “I’m afraid that if it continues like this, my knowledge gap will deepen”, and “I worry about not being able to graduate in time”. Since skill-oriented practice sessions take a significant part of the training curriculum of both universities, some of the respondents’ concerns were as such “I worry about I can’t learn practical skills properly”, “I can’t obtain enough experience in the procedure that deals with the patient”, and “My clinical training sessions were often canceled”. Many respondents worried about employment in the future: “It may be difficult to find a job in this situation because I have responsibility for family in the future”, “I worry about how much I learned from studying. Is it enough for my future work?”, “No place to work after graduation”, “If it continues under this situation I worry about how hard to find a job and keep wondering if the online class could let us—Freshly graduated students apply our knowledge to work effectively”, “My future is so uncertain”, and “I worry about not being able to study abroad after my graduation here”. The respondents’ worries are generally perceived as being uncertain: “I’m concerned that if this pandemic persists for a long time, I won’t be able to travel back home without first going through a lot of paperwork”, “I’m worried for my life, as I’m not sure for the future”, “When will I be able to go abroad? When will be a normal life without a mask”, “When COVID-19 will out of this world”, “I come from a poor family, my father is gone, my mother is old, I have a lot of responsibility, I am so worried about my future life”, “My future plan could be delayed”, etc.

**Figure 1:**
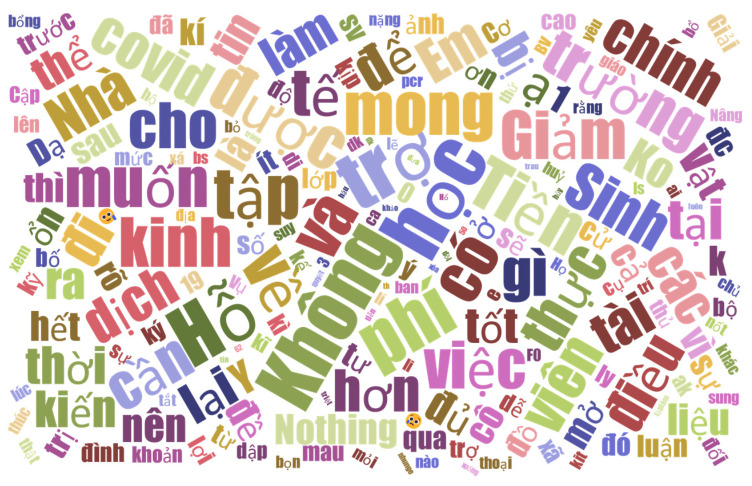
Need for support of the respondents in a word cloud (written in Vietnamese).

The respondents also mentioned any potential needs for support. Since most schooling is now done online, many have discussed the need for financial assistance, including cash grants, tuition exemptions, and scholarship amounts that might be modified to reflect rising living expenses. “I think we need at most financial support”. Some responders emphasized the necessity for rapid meals, immunizations, masks, alcohol, free exams, equipment, and dorm supplies. In addition to financial support, the majority of respondents mentioned their needs for online education infrastructure, such as better internet connections, better online services, facilities where students can borrow computers and tablets, and delivery of books and study materials to students’ homes. Additionally, the necessity of offering counseling and other forms of mental health care was brought up. In Figure 1, we show a word cloud to illustrate the most frequent words written in the Vietnamese language in the students’ opinions about the need for support. Among the most frequent words are the words such as “mong = wish”, “muốn = want”, “không = lack of”, “tiền = money”, “hỗ trợ = support”, “tài chính = financial”, “giảm = reducing”, “phí = fees”, “học = study”, “thực tập = practice”, “việc = job”, “trường = university”, “COVID”, “qua mau = quickly go”, “thực tế = reality”.

### 3.4. Conceptual Framework of COVID-19 Impacts on Students’ Campus Life

**Figure 2:**
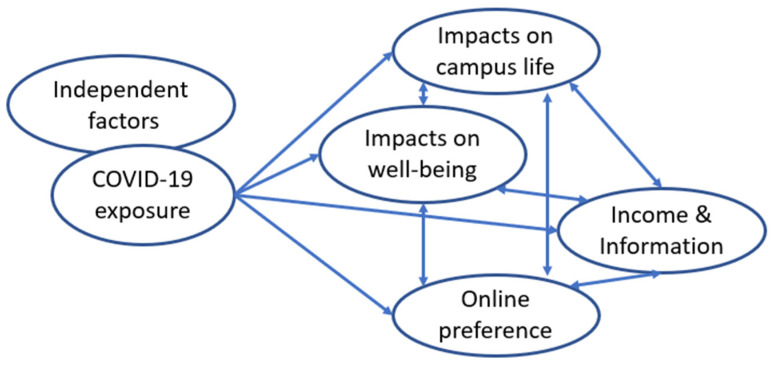
Proposed conceptual framework on impacts on well-being and online preference.

Based on the above results, we propose a model of the relationship between independent individual factors and the dependent variables, including perceived impacts on well-being, perceived impacts on campus life, and online preference (Figure 2).

Under “individual factors”, we include factors that we found significantly correlated with perceived personal well-being, such as urban universities, local students, female students, and students having pre-COVID-19 campus life experience. Under “impacts on well-being”, we include three dependent variables of perceived health impact, perceived stress, and worries about the future. The appropriateness of this group for the data set was checked by EFA (Kaiser–Meyer–Olkin 0.630 > 0.5, Bartlett’s Test of Sphericity’s *p* < 0.001, Principal Component Analysis showed Eigenvalues 1.856 > 1 for 1 component with Total Variance Explained 61.875 > 50, Component Matrix showed all three Factors’ loading > 0.7 as shown in Table 5).

Under “impacts on campus life”, “online preference”, and “income and information”, we include dependent variables of perceived impact on daily life, perceived impact on meals and shopping, perceived impact on taking classes, perceived impact on doing research, life plan change, income change, access to COVID-19 information, perceived satisfaction of online classes, and the proportion of online preference. The appropriateness of this group for the data set was checked by EFA (Kaiser–Meyer–Olkin 0.740 > 0.5, Bartlett’s Test of Sphericity’s *p* < 0.001, Principal Component Analysis showed Eigenvalues >1 for 3 components with Total Variance Explained 56.717 > 50, Component Matrix showed all Factors’ loading > 0.5 as shown in Table 5).

## 4. Discussion

The authors of this study looked into how the pandemic affected students’ health as well as other facets of campus life at two institutions in Vietnam. The survey was done two years after the outbreak, while the new fifth wave was still at its peak, and the students had already seen four epidemic waves from 2020 to early 2022. Except for a few occasions when the campus was temporarily available for in-person classes for a few months over this two-year period, the target universities’ students’ education was largely performed online. So far, we have published several reports regarding the impact of COVID-19 on the well-being and satisfaction of online classes among university students. We have found that pandemic impacts vary depending on the timing, location, characteristics of respondents, and so forth. In general, the results of this study show consistency with our previous reports [26,27,30,31].

The impact of COVID-19 on university students’ mental health and well-being is well described in the worldwide literature [3,7,32,33]. Regarding the impact on health, in this study, we found that a quarter of students reported a perceived impact on health, which was consistent with our previously reported data from three Asian universities that a third of the respondents had a perceived impact on health [27]. Regarding stress and mental well-being, our previous studies showed that over 50% of international students in Japan had been experiencing some kind of stress a year after the pandemic [30]. Moreover, factors such as students in higher grades and female students tend to perceive stress and concern about the future [27]. In the present study, we found similar numbers and patterns regarding stress and worry. Living in the dormitory is associated with perceived stress among students [27]; however, in this study, we have not found the same significant relationship.

University students are known to be susceptible to mental health issues. There has been evidence in many countries before the COVID-19 pandemic that stress, anxiety, and depression are progressively correlated with the years of university enrollment [3]. Our findings in this study have shown that the pandemic is probably an additional stressor to academic life but not an alleviating factor. We have shown that students’ pre-COVID-19 campus experiences have an impact on their well-being because they tend to perceive stress and worry about the future as being more intense. This is especially true for students who are in higher grades and have pre-COVID-19 campus experiences.

Academic satisfaction has been reported to be associated with well-being. A survey among students in Geneva at the early stage of the pandemic showed that lower academic satisfaction scores were significantly associated with stress [34]. Regarding satisfaction with online classes, we have shown that 61.9% of the students reported having some satisfaction with online classes. Regarding their opinion about the optimal proportion between online and face-to-face classes, over half of the students expressed a preference of 50% for online classes. This study shows higher online preference than one of our previous studies in Bulgaria, where more students prefer face-to-face classes [26]. We also confirmed in this report that students who expressed greater happiness with their online education might have felt less pressure, worry, and negative effects on their health. This outcome is consistent with the conclusions of our earlier report [27].

In relation to their pre-COVID-19 campus experience, the results have shown that students with pre-COVID-19 campus experience tend to show higher satisfaction with online classes than the students who had no such an experience. This group also shows a higher preference for 50% online classes, 80% online classes, and 100% online classes. The phenomenon could be explained by the fact that students who have experience studying in both pre-pandemic and in pandemic periods can compare the two modes and can recognize the merits of online education, while students who cannot compare tend to show more dissatisfaction with online education.

In this study, we have investigated factors that may associate with the perceived impact on the well-being of the respondents and have found a significant correlation between several factors and the well-being of respondents. Comparative studies have shown the different levels of impact on mental health across different universities and countries. A study about the mental health impact of university students in Canada and the UK has reported that 78.9% of students in Canada and 50.4% of students in the UK have reported worries about the future [35]. Our study has indicated a similar result, as 71.3% of students reported perceiving worries about the future, and this portion varies by university.

There is almost no comparative and conclusive data about differences in the well-being of university students in urban versus rural areas. A study in India has reported that the mental health of urban high school students is much better than the mental health of rural students [36]. A study in Bangladesh has reported that rural students were more likely to be significantly distressed than urban students [37]. In this study, we discovered that students at VNUA, a more urban area, have a tendency to view stress and worry about the future as being higher than students at TBUMP. This might be attributed to the higher percentage of students from rural areas at VNUA.

Regarding the impact on international students, an early study on the mental health impacts of international university students studying in the UK or USA has shown that 84.7% of students have perceived stress [38], which is much higher than the result of our study. However, there is a shortage of comparative data between international students and local students from the literature. Intriguingly, our research has shown that compared to local students, overseas students who have lived through the entire pandemic era tend to feel less impact on health and worry. What we found about worries for the future is similar to a study in the US which reported that many respondents feel uneasy about their plans for their continuing education and future perspective [39].

Gender also seems to play a role in mental health impact, but there is no conclusive evidence that being male or female is more susceptible. Many studies have reported that female students have worse emotional well-being compared to males [40]. A study in China has shown that being a female and coming from a rural area are risk factors for depressive traits among adolescent students [41]. However, a study in Korea has found that male students have higher mental health issues [42]. Our results show that female students tend to perceive more stress and worry about the future.

With regards to the relationship between student well-being and academic achievement, a study in Australia has shown significant associations between low well-being and an overall learning experience [43]. Our results show a consistent association between the impact of taking classes and doing research and the impact on well-being.

Ensuring that students can get a proper amount of pandemic information seems to be important. Overexposure to COVID-19-related information has been linked to psychological issues such as despair, anxiety, and insomnia, according to a study conducted in Thailand [17]. The current study has found a similar result that students who reported accessing more information on COVID-19 tended to perceive more stress and worry for the future.

In this study, we confirmed that students are in need of various kinds of support, including material, academic, campus life, and mental health support. A study conducted about a year after the COVID-19 outbreak has reported that university support provided by instructors and administration plays a mediating role in the relationship between the perceived impact of COVID-19 on graduation, future job prospects, and levels of student well-being [44]. We have found by qualitative analysis that universities need to continue to provide mental health support and consultation to meet the needs of students.

We realize that the Conceptual Framework of COVID-19 impacts on students’ well-being, campus life, and online class preference that we propose in Figure 2 is just a preliminary attempt to systematize our results while keep continuing to validate and investigate the new elements that may jump onto the stage. Taking the complexity, diversity, and fast-paced evolution of the issues at the global scale, especially on the aspects of online education, there are certainly many more influencing factors and determinants that may be involved.

The present study has limitations. It applies a cross-sectional study design, which is not able to compare the dynamics of pandemic impacts over time or to compare the effects by a control group; therefore, no causal inferences, such as the effects of the COVID-19 pandemic on students’ health, can be made. The respondents are limited to undergraduate students from two sites in northern Vietnam, which may hinder the generalizability of the results. Furthermore, the respondents may have recall bias. These limitations imply the need for further investigation to identify causal relationships and improve representativeness. Moreover, although the current study has proposed a preliminary framework on impacts on well-being and online preference, due to technical constraints, the authors were unable but may need to finalize and validate the model by applying model fit indices such as CFI and TLI for confirmatory factor analysis and in future studies.

## 5. Conclusions

The present study examines the perceived effects of the pandemic on well-being and the factors that may associate with online class preference among university students as well as investigate the need of students for support. The results from quantitative data have shown that impacts on mental aspects of well-being have appeared to be higher than the impact on health in general, especially since over 70% of respondents have reported worries about the future. Almost two third of the respondents reported having satisfaction with online classes, while over half of them preferred a program of 50% online classes. Students with pre-COVID-19 campus experience tend to report a higher impact on well-being, but they also tend to show higher satisfaction and higher preference for online classes. We also propose a conceptual framework for the relationship between independent factors such as university, gender, living alone status, factors related to impacts on well-being, factors related to impacts on campus life, factors related to online class preference, and income and access to information.

The results of this study show implications for university managers and educators to consider strategies addressing students’ well-being and post-pandemic online education. Providing support to university students to improve their resilience against the impact on their studying, campus life, health, and well-being should be prioritized during and post-pandemic. Strategies specifically targeting students at urban universities, who may live far away from home, female students, students having pre-COVID-19 campus life experience, and students with decreased income are probably in need of more support. The results of our study indicate that it is essential to give students support in order to increase the academic quality of classes, practice more, and create the conditions for conducting research according to the study subject’s requirements. Additionally, there is a need for career support, infrastructural support, health and mental health support, health support, and support for campus life. Students need to be advised not to be overwhelmed with information about the pandemic. The support could be focused on strengthening individual resilience and creating responsive environments. The results also have demonstrated that there is a high preference for online education as a form of education in the future. These findings also imply the need for further investigation of the future form of online learning, the factors that may predict the prolonged impact of the pandemic on students, and the role of supportive factors in alleviating the impacts of the pandemic.

## Figures and Tables

**Table 1:** Respondents’ characteristics.

Variable	Value	Total (*n* = 1598)	
		*n*	%
University	VNUA	291	18.2%
	TBUMP	1307	81.8%
Gender	Female	1120	70.1%
	Male	478	29.9%
Year of enrollment	First year	284	17.8%
	Second year	38	2.4%
	Third year	258	16.1%
	Fourth year	473	29.6%
	Fifth year	418	26.2%
	Sixth year	127	7.9%
Pre-COVID-19 campus experience	No (first year and second year)	322	20.2%
	Yes (third year and above)	1276	79.8%
Foreign student	Foreign student	61	3.8%
	Local student	1536	96.2%
Living status	Alone	594	37.2%
	With family	338	21.2%
	With roommate	666	41.7%
Living place	Dormitory	235	14.7%
	Rental	1092	68.3%
	Home	271	17.0%

**Table 2:** Perceived impacts on well-being in relation to the pre-COVID-19 campus experience.

Variable	Value	Pre-COVID-19 Campus Experience						Pearson Chi-Square	
		No (*n* = 322)		Yes (*n* = 1276)		Total (*n* = 1598)		Value (df)	*p*-Value
		*n*	%	*n*	%	*n*	%		
Perceived impact on health	Not at all	103	32.0%	257	20.1%	360	22.5%	24.357 (3)	0.000 ***
	Not so much	153	47.5%	688	53.9%	841	52.6%		
	Yes, some	55	17.1%	302	23.7%	357	22.3%		
	Yes, a lot	11	3.4%	29	2.3%	40	2.5%		
Perceived stress	Not at all	63	19.6%	158	12.4%	221	13.8%	15.563 (3)	0.001 **
	Not so much	133	41.3%	542	42.5%	675	42.2%		
	Yes, some	100	31.1%	498	39.0%	598	37.4%		
	Yes, a lot	26	8.1%	78	6.1%	104	6.5%		
Perceived worries	Not at all	20	6.2%	73	5.7%	93	5.8%	0.430 (3)	0.934
	Not so much	77	23.9%	288	22.6%	365	22.8%		
	Yes, some	157	48.8%	638	50.0%	795	49.7%		
	Yes, a lot	68	21.1%	277	21.7%	345	21.6%		

** *p* < 0.01; *** *p* < 0.001.

**Table 3:** Satisfaction and preference of online classes in relation to the pre-COVID-19 campus experience.

Variable	Value	Pre-COVID-19 Campus Experience						Pearson Chi-Square	
		No (*n* = 322)		Yes (*n* = 1276)		Total (*n* = 1598)		Value (df)	*p*-Value
		n	%	n	%	n	%		
Online class satisfaction	Not at all	12	3.7%	52	4.1%	64	4.0%	17.326 (3)	0.001 **
	Not so much	141	43.8%	404	31.7%	545	34.1%		
	Yes, some	145	45.0%	719	56.3%	864	54.1%		
	Yes, a lot	24	7.5%	101	7.9%	125	7.8%		
Online class preference	0%	15	6.7%	53	4.7%	68	5.0%	15.362 (4)	0.004 **
	20%	67	29.8%	222	19.6%	289	21.3%		
	50%	100	44.4%	583	51.4%	683	50.2%		
	80%	29	12.9%	204	18.0%	233	17.1%		
	100%	14	6.2%	73	6.4%	87	6.4%		

** *p* < 0.01.

**Table 4:** Association between well-being-related variables and other factors.

Variable		Health	Stress	Worry for Future
University	Pearson Chi-Square (df)	5.271 (3)	43.912 *** (3)	28.700 *** (3)
	Sig. (2-sided)	0.153	0.000	0.000
	*n*	1598	1598	1598
Foreign student	Pearson Chi-Square (df)	15.568 ** (3)	3.755 (3)	12.581 ** (3)
	Sig. (2-sided)	0.001	0.289	0.006
	*n*	1597	1597	1597
Female	Pearson Chi-Square (df)	12.607 ** (3)	18.259 *** (3)	39.284 *** (3)
	Sig. (2-sided)	0.006	0.000	0.000
	*n*	1598	1598	1598
Year of enrollment	Pearson Chi-Square (df)	54.396 *** (15)	36.203 ** (15)	17.505 (15)
	Sig. (2-sided)	0.000	0.002	0.290
	*n*	1598	1598	1598
Pre-COVID-19 campus experience	Pearson Chi-Square (df)	24.357 *** (3)	15.563 ** (3)	0.430 (3)
	Sig. (2-sided)	0.000	0.001	0.934
	*n*	1598	1598	1598
Living status (alone, with family, or roommates)	Pearson Chi-Square (df)	12.933 * (6)	19.143 ** (6)	2.919 (6)
	Sig. (2-sided)	0.044	0.004	0.819
	*n*	1598	1598	1598
Living place (dormitory, rental place, home)	Pearson Chi-Square (df)	8.631 (6)	11.674 (6)	4.295 (6)
	Sig. (2-sided)	0.195	0.070	0.637
	*n*	1598	1598	1598
Impact on taking classes	Correlation Coefficient	0.301 **	0.313 **	0.281 **
	Sig. (2-tailed)	0.000	0.000	0.000
	*n*	1598	1598	1598
Online class satisfaction	Correlation Coefficient	−0.058 *	−0.075 **	−0.028
	Sig. (2-tailed)	0.021	0.003	0.270
	*n*	1598	1598	1598
Online class preference	Correlation Coefficient	−0.039	−0.091 **	−0.090 **
	Sig. (2-tailed)	0.150	0.001	0.001
	*n*	1360	1360	1360
Impact on research	Correlation Coefficient	0.329 **	0.316 **	0.252 **
	Sig. (2-tailed)	0.000	0.000	0.000
	*n*	1598	1598	1598
Impact on meal and shopping	Correlation Coefficient	0.487 **	0.366 **	0.235 **
	Sig. (2-tailed)	0.000	0.000	0.000
	*n*	1598	1598	1598
Impact on daily life	Correlation Coefficient	0.489 **	0.480 **	0.395 **
	Sig. (2-tailed)	0.000	0.000	0.000
	*n*	1598	1598	1598
Income change	Correlation Coefficient	−0.054 *	−0.052 *	−0.080 **
	Sig. (2-tailed)	0.031	0.037	0.001
	*n*	1598	1598	1598
Life plan change	Correlation Coefficient	0.330 **	0.374 **	0.435 **
	Sig. (2-tailed)	0.000	0.000	0.000
	*n*	1598	1598	1598
Access to information	Correlation Coefficient	−0.012	0.112 **	0.217 **
	Sig. (2-tailed)	0.622	0.000	0.000
	*n*	1598	1598	1598

* *p* < 0.05; ** *p* < 0.01; *** *p* < 0.001.

**Table 5:** Factor loadings by components.

Factor Loadings	Impacts on Well-Being	Impacts on Campus Life	Online Preference	Income and Information
Perceived stress	0.850			
Worry about future	0.773			
Perceived health impact	0.773			
Daily life		0.799		
Meals and shopping		0.718		
Research		0.662		
Taking classes		0.645		
Life plan change		0.636		
Online satisfaction			0.790	
Best amount online			0.684	
Income change				0.715
Access to information				0.592

## Data Availability

The data presented in this study are not publicly available.
